# Suppression of nuclear factor-κB activity in macrophages by chylomicron remnants: modulation by the fatty acid composition of the particles

**DOI:** 10.1111/j.1742-4658.2009.07260.x

**Published:** 2009-10

**Authors:** Clara De Pascale, Valerie Graham, Robert C Fowkes, Caroline P D Wheeler-Jones, Kathleen M Botham

**Affiliations:** Department of Veterinary Basic Sciences, The Royal Veterinary CollegeLondon, UK

**Keywords:** chylomicron remnants, dietary fats, inflammatory cytokines, macrophage foam cells, nuclear factor-κB

## Abstract

Current evidence indicates that chylomicron remnants (CMR) induce macrophage foam cell formation, an early event in atherosclerosis. Inflammation also plays a part in atherogenesis and the transcription factor nuclear factor-κB (NF-κB) has been implicated. In this study, the influence of CMR on the activity of NF-κB in macrophages and its modulation by the fatty acid composition of the particles were investigated using macrophages derived from the human monocyte cell line THP-1 and CMR-like particles (CRLPs). Incubation of THP-1 macrophages with CRLPs caused decreased NF-κB activation and downregulated the expression of phospho-p65–NF-κB and phospho-IκBα (pIκBα). Secretion of the inflammatory cytokines tumour necrosis factor α, interleukin-6 and monocyte chemoattractant protein-1, which are under NF-κB transcriptional control, was inhibited and mRNA expression for cyclooxygenase-2, an NF-κB target gene, was reduced. CRLPs enriched in polyunsaturated fatty acids compared with saturated or monounsaturated fatty acids had a markedly greater inhibitory effect on NF-κB binding to DNA and the expression of phospho-p65–NF-κB and pIκB. Lipid loading of macrophages with CRLPs enriched in polyunsaturated fatty acids compared with monounsaturated fatty acids or saturated fatty acids also increased the subsequent rate of cholesterol efflux, an effect which may be linked to the inhibition of NF-κB activity. These findings demonstrate that CMR suppress NF-κB activity in macrophages, and that this effect is modulated by their fatty acid composition. This downregulation of inflammatory processes in macrophages may represent a protective effect of CMR which is enhanced by dietary polyunsaturated fatty acids.

## Introduction

Atherosclerosis is initiated by the entry of lipoproteins into the artery wall which stimulates proinflammatory events in the endothelium. This condition is a systemic ‘response-to-injury reaction’ in which monocytes/macrophages play an essential role [1]. Monocytes are recruited by the proinflammatory signals and transmigrate into the subendothelial space where they differentiate into tissue macrophages and take up lipoproteins, eventually becoming so engorged with lipids that they form foam cells, which are characteristic of early atherosclerotic lesions [2].

Extensive studies have established that low-density lipoprotein, particularly after oxidation, plays a major role in foam cell formation and atherogenesis [3]. There is, however, considerable evidence to support the idea that chylomicron remnants (CMR), the lipoproteins which carry dietary lipids from the gut to the liver, are also proatherogenic [4]. Thus, CMR are taken up by and retained in the artery wall [5], remnant-like particles have been found in human aortic *intima* and atherosclerotic plaque [6,7], and delayed clearance of CMR from the circulation is associated with atherosclerosis development [8,9]. Furthermore, we and others have demonstrated that CMR cause foam cell formation in human monocyte-derived macrophages and in macrophage cell lines [10–12].

The induction of foam cell formation by CMR is clearly an atherogenic response; however, atherosclerosis is not only a disorder of lipid accumulation, but is also recognized as an inflammatory disease [13]. Nuclear factor-κB (NF-κB) is a major transcription factor involved in inflammatory responses in a number of cell types and plays a key role in atherosclerosis [14]. The NF-κB family consists of five members, p65 (RelA), cRel, RelB, NF-κB1 (p50 and its precursor p105) and NF-κB2 (p52 and its precursor p100), which can form either homodimers or heterodimers, but the most abundant and well-studied complex is p65/p50 [15]. The activated form of p65–NF-κB is not usually expressed in normal vessels, but is present in atherosclerotic lesions, and NF-κB-dependent genes are induced in the disease process [16]. Moreover, it is well established that NF-κB controls the transcription of a range of genes important for regulating inflammatory events in macrophages, including the expression of proinflammatory cytokines and chemokines [e.g. tumour necrosis factor α (TNFα), interleukin (IL)-1β, IL-6, monocyte chemoattractant protein-1 (MCP-1)] and the enzyme cyclooxgenase-2 (COX-2) [17,18]. NF-κB dimers are inactive when bound to the endogenous inhibitory protein IκB and although several isoforms of IκB exist, the most predominant is IκBα [15]. Phosphorylation of IκB by upstream kinases results in its Lys48-linked polyubiquitylation and degradation, permitting translocation of active NF-κB to the nucleus and transcriptional regulation of NF-κB-dependent target genes [19,20].

Oxidized low density lipoprotein (oxLDL) can suppress NF-κB activity in macrophages [21] and there is some evidence for its involvement in oxLDL-induced macrophage foam cell formation. Uptake of oxLDL is inhibited in activated p50-deficient murine macrophages [22], and in a recent study, reduced lipid loading in response to oxLDL was observed in macrophages overexpressing a degradation-resistant IkBα, an effect that was attributed to increased cholesterol efflux [23]. Little is known, however, about the effects of CMR on NF-κB activity in macrophages.

The composition of the diet is known to be important in the development of atherosclerosis [24–26], and a major dietary determinant is the amount and type of fat present. It is well established that consumption of saturated fats (SFA) is associated with increased risk of atherosclerosis development, whereas intake of monounsaturated fats (MUFA) and polyunsaturated fats (PUFA) of both the n-6 and n-3 series is beneficial [26,27]. In previous studies, we have shown that the fatty acid composition of CMR reflects that of the diet [28] and modulates their clearance from the blood by the liver [29]. Furthermore, our recent work has established that the fatty acid composition of chylomicron remnant-like particles (CRLPs) markedly influences their induction of macrophage foam cell formation. In these studies, we found that CRLPs enriched in SFA are taken up more rapidly and cause greater lipid accumulation in macrophages than those enriched in n-6 or n-3 PUFA [30]. These findings provide strong evidence that induction of macrophage foam cell formation is influenced by dietary fatty acids during their transport from the gut to the liver in CMR in the postprandial phase.

In this study, we investigated the effects of CMR on NF-κB activation in macrophages and determined whether these are modulated by the fatty acid composition of the particles. CRLPs enriched in SFA, MUFA, n-6 PUFA or n-3 PUFA prepared using triacylglycerol derived from palm, olive, corn or fish oil, respectively, and macrophages derived from the human monocyte cell line THP-1 were used as the experimental model. The influence of CRLPs on processes regulated by NF-κB, including chemokine secretion, COX-2 expression and cholesterol efflux were also examined.

## Results

### Effect of CRLPs on NF-κB activation in macrophages

**Fig. 1:**
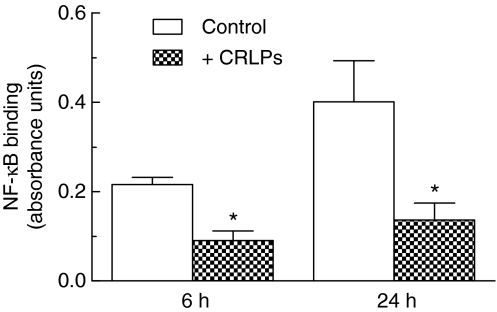
THP-1 macrophages were incubated with or without CRLPs containing n-6 PUFA (trilinolein) (0.29 μmol triacylglycerol·mL^−1^) for 6 or 24 h and NF-κB binding was measured using an ELISA based kit (TransAM). Data are the mean of three separate experiments and error bars show the SEM. *P < 0.05 versus corresponding control.

**Fig. 2:**
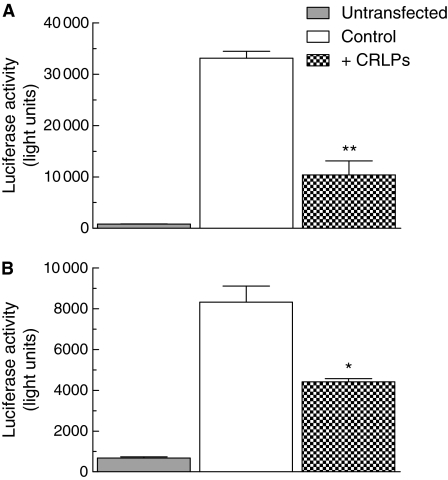
THP-1 macrophages transfected with the pNF-κB Luc reporter gene construct were incubated with or without (control) CRLPs containing n-6 PUFA (trilinolein) (0.29 μmol triacylglycerol·mL^−1^) (A) or corn CRLPs (0.30 mmol triacylglycerol·mL^−1^) (B) for 8 h and NF-κB activity was determined using a luciferase assay. Nontransfected cells were also assayed for comparison. Data shown are the mean from three replicate incubations and error bars show the SEM. *P < 0.05, **P < 0.01 versus control.

Activation of NF-κB releases NF-κB dimers which translocate to the nucleus where they bind to specific DNA nucleotide sequences to modulate the expression of target genes [14]. Thus, binding to DNA consensus sites can be used as a measure of NF-κB activity. Initial experiments using a p65–NF-κB DNA-binding ELISA-based assay showed that incubation of CRLPs (containing triacylglycerol enriched in n-6 PUFA from corn oil) with THP-1 macrophages for 6 or 24 h resulted in a highly significant reduction in NF-κB activation compared with that found in control cells incubated in the absence of CRLPs (% control value, *n* = 3: 6 h, 40.2 ± 8.3, *P* < 0.001; 24 h, 29.3 ± 7.3, *P* < 0.001) (Fig. 1). Inhibition of NF-κB transcriptional activity by CRLPs containing trilinolein or triacylglycerol from corn oil was confirmed by measuring luciferase activity in cells transfected with the pNF-κB Luc plasmid (Fig. 2).

### Effects of CRLPs on cytokine and chemokine secretion and mRNA expression in macrophages

**Fig. 3:**
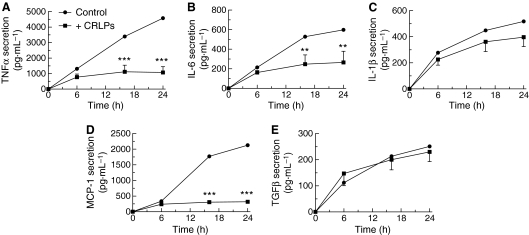
THP-1 macrophages were incubated with or without (control) CRLPs containing n-6 PUFA (trilinolein) (0.29 μmol triacylglycerol·mL^−1^) for 6, 16 or 24 h and the secretion of (A) TNFα, (B) IL-6, (C) IL-1β, (D) MCP-1 and (E) TGFβ was determined by ELISA. Data are the mean of three (IL-6), four (TNFα, MCP-1) or five (IL-1 β) separate experiments normalized to the average control value at each time point. Error bars show the SEM. **P < 0.01, ***P < 0.001 versus control.

We initially examined the effects of CRLPs on the release of TNFα, IL-6, IL-1β and MCP-1, which are under NF-κB transcriptional control [31–34], and of transforming growth factor β (TGFβ) whose synthesis is NF-κB independent [35] (Fig. 3). In THP-1 macrophages exposed to CRLPs prepared with triacylglycerol containing n-6 PUFA (trilinolein) there was a marked reduction in IL-6, TNFα and MCP-1 secretion compared with controls over 24 h, and analysis by two-way ANOVA indicated that, taking into account all three time points tested, the changes were statistically significant (IL-6, *P* < 0.05; TNFα, MCP-1, *P* < 0.01) (Fig. 3A,B,D). At individual time points, significant downregulation of TNFα (Fig. 3A), MCP-1 (Fig. 3D) (*P* < 0.001) and IL-6 (Fig. 3B) (*P* < 0.01) secretion was observed after 16 and 24 h (*P* < 0.001). IL-1β secretion also showed a tendency to decrease after CRLP treatment, but in this case the changes did not reach significance (Fig. 3C). By contrast, CRLPs had no effect on the secretion of TGFβ at any of the time points assessed (Fig. 3E).

**Fig. 4:**
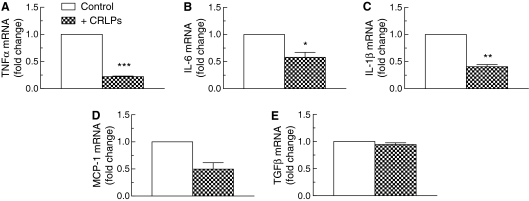
THP-1 macrophages were incubated with or without (control) CRLPs containing n-6 PUFA (trilinolein) (0.29 μmol triacylglycerol·mL^−1^) for 16 h and the abundance of mRNA transcripts for (A) TNFα, (B) IL-6, (C) IL-1β, (D) MCP-1 and (E) TGFβ was determined using quantitative real-time PCR. Data were normalized using the values obtained for GAPDH and are the mean from three separate experiments. Error bars show the SEM. *P < 0.05, **P < 0.01, ***P < 0.001 versus control (Student’s *t*-test).

The abundance of mRNA transcripts for each of the cytokines was determined after incubation of THP-1 macrophages with CRLPs for 16 h, and the results are shown in Fig. 4. There was a marked decrease in mRNA levels for TNFα (−78%, *P* < 0.001) (Fig. 4A), IL-6 (−42%, *P* < 0.05) (Fig. 4B), IL-1β (−59%, *P* < 0.01) (Fig. 4C) and MCP-1 (−50%, *P* = 0.051) (Fig. 4D), although TGFβ mRNA concentrations were unaffected (Fig. 4E).

### Effect of the fatty acid composition of CRLPs on NF-κB activation in macrophages

**Fig. 5:**
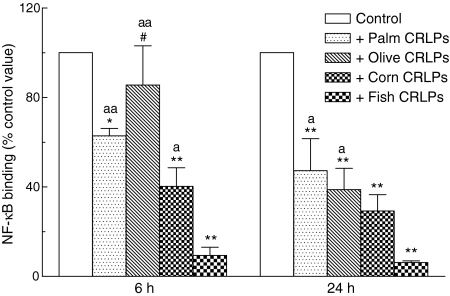
THP-1 macrophages were incubated with or without (control) palm, olive, corn or fish CRLPs (0.3 μmol triacylglycerol·mL^−1^) for 6 or 24 h and NF-κB binding was measured using an ELISA based kit (TransAM). Data are expressed as % control value at each time point and are the mean of three separate experiments. Error bars show the SEM. *P < 0.05; **P < 0.001 versus control; #*P* < 0.01 versus corn CRLPs; ^a^*P* < 0.05; ^aa^*P* < 0.001 versus fish CRLPs.

The p65–NF-κB DNA-binding ELISA (TransAM™) was used to assess the influence of CRLPs on NF-κB activation. THP-1 macrophages were incubated with palm, olive, corn or fish CRLPs (enriched with SFA, MUFA, n-6 PUFA and n-3 PUFA, respectively) and the data expressed as % control at each time point are shown in Fig. 5. There was no significant difference between the control values obtained at 6 (0.216 ± 0.016) and 24 h (0.401 ± 0.092). Analysis by two-way ANOVA indicated that, taking into account both time points, NF-κB binding was decreased by all four types of CRLPs (*P* < 0.01), with significant decreases (versus control) evident with palm (*P* < 0.05), corn (*P* < 0.001) and fish (*P* < 0.001), but not olive CRLPs at 6 h, and with all types of particles after 24 h (*P* < 0.001). Comparing the various types of CRLPs, NF-κB binding was decreased to a greater extent by fish CRLPs than by palm, olive or corn CRLPs, whereas corn CRLPs caused increased inhibition in comparison with olive CRLPs. At individual time points, fish CRLPs had a markedly greater inhibitory effect (reaching −94% at 24 h) than palm or olive CRLPs after 6 h (*P* < 0.001) and 24 h (*P* < 0.01), and also compared with corn CRLPs at 6 h (*P* < 0.05). In addition, macrophages treated with corn compared with olive CRLPs showed lower NF-κB binding after 6 h incubation (*P* < 0.01). These results indicate that the inhibitory effect of CRLPs on NF-κB activation is influenced by the fatty acid composition of the particles.

**Fig. 6:**
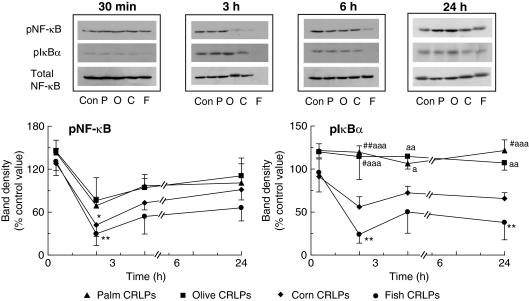
THP-1 macrophages were incubated with or without (con) palm (P), olive (O) corn (C) or fish (F) CRLPs (0.3 μmol triacylglycerol·mL^−1^) for the times indicated and the expression of phosphorylated p65–NF-kB (pNF-κB), phosphorylated IκBα (pIκBα) and total NF-κB determined by immunoblotting. The upper panels show representative immunoblots from a single experiment. The lower panels show densitometric analyses of immunoblots from three (pIκBα) or four (pNF-κB) individual experiments. Data were normalized to total NF-κB expression and are expressed as % control value at each time point. Error bars show the SEM. *P < 0.05, **P < 0.01 versus control; #*P* < 0.05, ##*P* < 0.01 versus corn CRLPs; ^a^*P* < 0.05, ^aa^*P* < 0.01, ^aaa^*P* < 0.001 versus fish CRLPs.

Phosphorylation of p65–NF-κB plays a critical role in regulating its transcriptional activity. To further investigate the effects of the fatty acid composition of CRLPs on NF-κB activation, phosphorylation of p65–NF-κB (Ser536) was evaluated by immunoblotting after incubation of THP-1 macrophages with palm, olive, corn or fish CRLPs (0.5–24 h) (Fig. 6). Although levels of phospho-p65–NF-κB are usually low in normal cells, we found relatively high expression in control macrophages. Our control cells, however, are likely to be partially activated because of their exposure to phorbol ester during differentiation into macrophages. Phospho-p65–NF-κB expression was suppressed to different extents by CRLPs depending on the fatty acid composition of the particles. This inhibitory effect was confirmed by densitometric analyses of immunoblots from four separate experiments, which indicated significant reductions in the level of phospho-p65–NF-κB after 3 h incubation with corn and fish CRLPs, but not palm and olive CRLPs (Fig. 6). NF-κB activity in control samples did not vary significantly over the time-course examined.

**Fig. 7:**
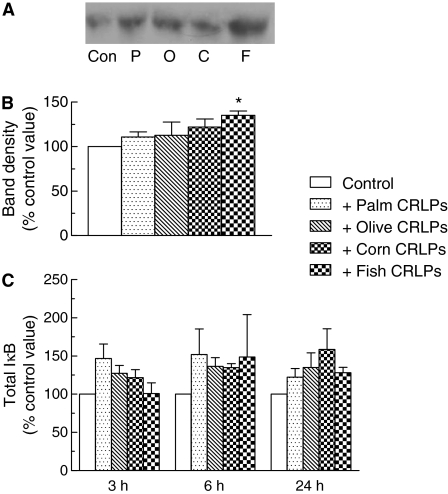
THP-1 macrophages were incubated with or without (con) palm (P), olive (O), corn (C) or fish (F) CRLPs (0.3 μmol triacylglycerol·mL^−1^) for 3, 6 or 24 h and the total IκBα content of the cells was determined by immunoblotting (3 h only shown; A, a representative immunoblot; B, densitometric analysis) or using an ELISA kit (C). Data shown are the mean from three separate experiments and error bars show the SEM. Immunoblotting data were normalized by equal protein loading (80 μg protein·lane^−1^). ELISA data are shown as % control value; the absolute control values did not change significantly with time (absorbance units: 3 h, 0.56 ± 0.14; 6 h 0.53 ± 0.06; 24 h, 0.37 ± 0.06). *P < 0.05 versus control.

In the canonical NF-κB pathway, activation of the IκB kinase complex leads to phosphorylation and subsequent degradation of IκBα, thus allowing translocation of NF-κB to the nucleus [20]. To determine whether modulation of IκBα serine phosphorylation status plays a part in mediating the inhibitory effects of CRLPs on NF-κB activity, their influence on expression of phosphorylated IκBα (pIκBα) was assessed (Fig. 6). In keeping with their effects on NF-κB phosphorylation and activity, CRLPs caused a downregulation of pIκBα expression which was dependent on their fatty acid composition, with corn and fish CRLPs having a greater effect than palm and olive CRLPs. Again, macrophages treated with fish CRLPs showed the strongest reduction in expression, with protein levels being significantly lower than in control cells after 3 and 24 h and palm or olive CRLP-treated macrophages at all time points except 0.5 h. In addition, pIκBα expression was decreased after treatment of macrophages with corn CRLPs compared with palm CRLPs at 3 and 24 h and compared with olive CRLPs at 3 h. Total IκBα levels, as assessed by immunoblotting, were significantly increased by fish CRLPs, but not palm olive or corn CRLPs (*P* < 0.05, Fig. 7A,B) after 3 h incubation, and no significant changes were observed with any of the four types of CRLPs at the other time points tested (data not shown).

The total IκBα content of THP-1 macrophages after treatment with palm, olive, corn or fish CRLPs for 3, 6 and 24 h was also determined by ELISA and the results are shown in Fig. 7B. Data are expressed as % control value (control values at the three time points were not significantly different). Total IκBα levels were not significantly changed by any of the four types of CRLPs.

### Effect of the fatty acid composition of CRLPs on COX-2 mRNA expression

**Fig. 8:**
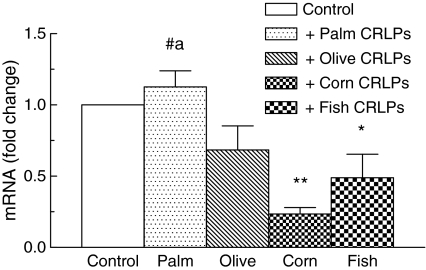
THP-1 macrophages were incubated with or without (control) palm, olive, corn or fish CRLPs (0.3 μmol triacylglycerol·mL^−1^) for 24 h and the abundance of mRNA transcripts for COX-2 was determined by quantitative real-time PCR. Data were normalized using the values obtained for GAPDH and are the mean from 11 separate experiments. Error bars show the SEM. *P < 0.05, **P < 0.001 versus control; #*P* < 0.001 versus corn CRLPs; ^a^*P* < 0.01 versus fish CRLPs.

The effect of CRLPs of varying fatty acid composition on expression of COX-2, an NF-κB target gene [40] was evaluated by determining mRNA levels for the enzyme by quantitative real-time PCR after 24 h incubation with palm, olive, corn or fish CRLPs. As shown in Fig. 8, treatment of THP-1 macrophages with corn or fish CRLPs significantly decreased COX-2 mRNA levels when compared with controls or with cells treated with palm CRLPs (corn CRLPs versus control and palm CRLPs, *P* < 0.001; fish CRLPs versus control, *P* < 0.05, versus palm CRLPs *P* < 0.01).

### Cholesterol efflux from THP-1 macrophages is modulated by the fatty acid composition of CRLPs

**Fig. 9:**
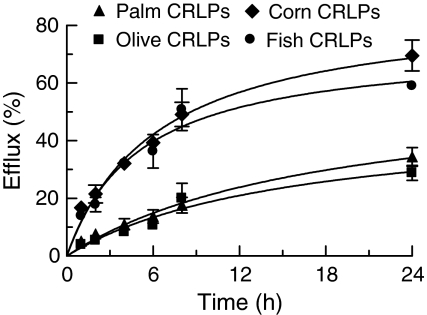
THP-1 macrophages were incubated with palm, olive, corn, or fish CRLPs (30 μg cholesterol·mL^−1^) radiolabelled in cholesterol (4 KBq [^3^H]cholesterol·mL^−1^, 52.4 KBq·μmol^−1^) for 48 h. The medium containing lipoproteins was then removed and the incubation was continued for 24 h in the presence of apoA-I/phosphatidylcholine (100 μg·mL^−1^). Data are expressed as a percentage of the total radioactivity in the cells at the end of the loading period (time 0) and are the mean of three separate experiments. Error bars show the SEM. The efflux curves were significantly different (two-way ANOVA) as follows: palm CRLPs versus corn CRLPs, *P* < 0.001, versus fish CRLPs, *P* < 0.01; olive CRLPs versus corn and fish CRLPs, *P* < 0.001.

Because inhibition of NF-κB activation has previously been linked to increased cholesterol efflux activity [23], the effects of CRLPs of varying fatty acid composition on cholesterol efflux from macrophages were determined. As shown in Fig. 9, the rate of efflux of radioactivity was markedly faster in macrophages treated with corn or fish CRLPs compared with palm or olive CRLPs (palm CRLPs versus corn CRLPs, *P* < 0.001, versus fish CRLPs, *P* < 0.01; olive CRLPs versus corn and fish CRLPs, *P* < 0.001).

## Discussion

Although there is now substantial evidence to indicate that CMR cause macrophage foam cell formation without prior oxidation [4,10–12], little is known about the influence of these particles on macrophage inflammatory functions and how this relates to their induction of lipid accumulation. The study presented here provides evidence that CMR downregulate NF-κB activation, that this is accompanied by modulation of inflammatory processes in macrophages, and that the extent of the inhibitory action on the NF-κB pathway depends upon the fatty acid composition of the particles.

Because it is difficult to obtain CMR from human blood uncontaminated with lipoproteins of a similar density such as chylomicrons and very low density lipoprotein, we used model CRLPs containing human apolipoprotein E (apoE). We and others have shown previously that these particles have a size, density and lipid composition [11] in the range of physiological CMR [36], and our work has demonstrated that they cause lipid accumulation in macrophages to an extent which is comparable with that observed with rat CMR in J774 macrophages [11,12].

Our initial experiments clearly showed that NF-κB binding to DNA is downregulated by CRLPs in THP-1 macrophages (Fig. 1) and further experiments using an NF-κB luciferase reporter gene construct assay confirmed that the particles inhibit NF-κB transcriptional activity in these cells (Fig. 2). This conclusion is further supported by our studies evaluating the effects of CRLPs on cytokine/chemokine secretion by macrophages. TNFα stimulates NF-κB activity [31] and its promoter also contains NF-κB binding sites causing positive autoregulation [37], whereas IL-6, IL-1β and MCP-1 are all under NF-κB transcriptional control [32–34]. The anti-inflammatory cytokine, TGFβ, however, is not controlled by NF-κB-dependent mechanisms. Thus, our findings that the secretion of TNFα, IL-6 and MCP-1 by THP-1 macrophages were all strongly downregulated by CRLPs, whereas TGFβ release was unaffected (Fig. 3), is in keeping with the reduced level of NF-κB activation following CRLP treatment. There has been little study of the effects of CRLPs on macrophage cytokine synthesis, but our results agree with those of a recent study reporting inhibition of TNFα secretion by CRLPs in primary human macrophages [38]. Further evidence that CMR inhibit NF-κB and that this is reflected in reduced transcriptional activity and modification of cytokine synthesis is provided by our mRNA expression studies, which clearly show parallel attenuation of TNFα, IL-6 and MCP-1 expression, but not TGFβ, in macrophages exposed to CRLPs (Fig. 4). Against this, we did not detect any significant decrease in the secretion of IL-1β, another cytokine under NF-κB control [33], after exposure of macrophages to CRLPs (Fig. 3). However, considerably less IL-1β was secreted compared with other cytokines (e.g. in control incubations after 24 h concentrations of IL-1β were ∼ 14% those of TNFα). Under these circumstances, it is likely to be more difficult to demonstrate a statistically significant effect and in fact, the mean values for the production of the cytokine were lower in CRLP-treated cells than in control cells at all time points. Furthermore, we detected a marked decrease in the expression of mRNA for IL-1β in macrophages treated with CRLPs compared with control cells (Fig. 4E), suggesting that the gene is downregulated at the transcriptional level. Overall, therefore, our results demonstrate that macrophage NF-κB activity is suppressed by CMR in macrophages and that cytokine expression is modified by the particles in a manner that correlates with NF-κB dependency.

Our findings contrast with those of one previous study by Okumura *et al.* [39], who reported that rat CMR increase IL-1β secretion and mRNA expression and enhance NF-κB binding to a consensus DNA binding probe in human THP-1 macrophages. However, because their study used lipoproteins and cells from non-homologous species together with semiquantitative analyses of mRNA levels and NF-κB binding, the results are not likely to be a reliable reflection of CMR effects on macrophages.

Our previous studies have established that the rate of uptake of CRLPs by THP-1 macrophages and their subsequent induction of foam cell formation differs, depending on their fatty acid composition, with SFA-enriched particles taken up more rapidly and causing more lipid accumulation than those enriched with n-6 PUFA and n-3 PUFA [30]. Thus, enrichment of CMR with SFA compared with PUFA may increase their atherogenicity. In this study, we investigated whether the differential effects of CRLPs of varying fatty acid composition on macrophages relate to their modulation of NF-κB activation. To prepare CRLPs of varying fatty acid composition, triacylglycerol derived from natural dietary oils was used, so that although the particles were enriched in SFA, MUFA, n-6 PUFA or n-3 PUFA (using triacylglycerol derived from palm, olive, corn or fish oil, respectively) they also contained a complex mixture of fatty acids which reflects the composition of the parent oils and of physiological CMR derived from them [28]. The triacylglycerol/total cholesterol ratio in the four types of CRLPs used for this study was similar (Table 1) and we have shown previously that they contain similar amounts of apoE [30]. Any differences in their effects on NF-κB activation and related processes, therefore, can be attributed directly to differences in their fatty acid composition.

**Table 1:** Lipid content of chylomicron remnant-like particles (CRLPs). CRLPs containing triacylglycerol (TG) from palm, olive, corn and fish or trilinolein were prepared as described in Materials and Methods and the TG and total cholesterol (TC) content (μmol·mL^−1^) was measured. Data shown are the mean ± SEM of six separate preparations.

CRLP	TG	TC	TG/TC
Palm	4.97 ± 1.44	0.72 ± 0.20	6.68 ± 0.74
Olive	5.23 ± 1.73	0.67 ± 0.17	7.49 ± 0.67
Corn	5.82 ± 1.11	0.72 ± 0.12	7.91 ± 0.53
Fish	5.76 ± 0.70	0.78 ± 0.16	7.87 ± 0.85
Trilinolein	6.56 ± 1.82	0.92 ± 0.26	7.53 ± 1.13

Treatment of macrophages with each of the four types of CRLPs resulted in reduced NF-κB activation, as determined by DNA binding, and this effect was clearly modulated by their fatty acid composition, with fish CRLPs causing the strongest inhibition (−94% after 24 h) followed by corn CRLPs (−70%), and palm and olive CRLPs (−53 to 61%) (Fig. 5). Expression of phospho-p65–NF-κB and pIκBα showed a similar pattern, with decreased levels of both proteins found in macrophages incubated with corn and fish CRLPs compared with palm and olive CRLPs (Fig. 6). These changes were not caused by decreases in total NF-κB (used to normalize the results) or decreases in total IκBα levels (Fig. 7), neither of which were significantly reduced by any of the CRLP types. Indeed, immunoblotting showed that there was a significant increase in total IκBα levels in macrophages treated with fish CRLPs for 3 h, corresponding to the strongest decrease in pIκBα concentrations observed (−75%) at any time point and with any CRLP type (Fig. 6). Because phosphorylation of IκBα targets it for degradation [20], these results are consistent with the findings on pIκBα levels (Fig. 6) and the decreased phosphorylation of the inhibitor will result in reduced NF-κB activation. Although NF-κB DNA binding was significantly reduced by palm and olive CRLPs (Fig. 5), whereas expression of phospho-p65–NF-κB and pIkBα was not (Fig. 6), it seems likely that this difference is because of the relative sensitivity of the two assays. Thus, CRLPs enriched in PUFA, and particularly n-3 PUFA, were more effective in downregulating NF-κB activity than those enriched in SFA or MUFA. Together, these results demonstrate that NF-κB activation is inhibited by exposure to CMR, and that the fatty acid composition of the particles modulates this effect.

Earlier studies on the effects of free fatty acids on NF-κB activity in macrophages have also suggested that different types of fatty acids have differential effects. Weldon *et al.* [40] demonstrated that the n-3 PUFAs eicosapentaenoic acid and docosahexaenoic acid, which are found in fish oil, downregulate LPS-induced NF-κB DNA binding and p65–NF-κB expression, and increase IκBα expression and docosahexaenoic acid and/or eicosapentaenoic acid have also been reported to suppress NF-κB activation induced by LPS, interferon-γ or receptor activator of NF-κB ligand (RANKL) [41–43]. By contrast, in experiments with murine macrophage cell lines, Fuhrmann *et al.* [44] did not detect an effect of n-6 PUFA (linoleic acid) or the n-3 PUFA α-linolenic acid on NF-κB activation, whereas SFA have been reported to enhance lipopolysaccharide-induced NF-κB activation [45]. These findings, therefore, are generally consistent with our results in that n-3 PUFA from fish oil exert a greater inhibitory effect on NF-κB activation than n-6 PUFA or SFA.

During inflammation, the NF-κB pathway increases COX-2 transcription and this is responsible for the prolonged biosynthesis of prostanoids [46]. This study shows that the expression of COX-2 mRNA in CRLP-treated macrophages is dependent on their fatty acid composition, with corn and fish, but not palm and olive CRLPs, promoting downregulation (Fig. 8). Because COX-2 is a target gene for NF-κB, these results provide further evidence that CMR enriched in PUFA compared with MUFA or SFA cause greater inhibition of NF-κB activation and suggest a possible down-stream effect of PUFA-enriched particles on prostaglandin production. Palm and olive CRLPs, however, did have an inhibitory effect on NF-κB binding and activation in the absence of any downregulation of COX-2 mRNA expression (Figs 5, 6 and 8), suggesting that fatty acids delivered to the cells in CMR differentially affect NF-κB activation and downstream gene expression.

We have previously shown that CRLPs enriched in SFA are taken up more rapidly by THP-1 macrophages than those enriched in n-6 or n-3 PUFA, and thus enhance foam cell formation [30]. Clearly, however, the amount of lipid accumulated depends on the balance between lipoprotein uptake and subsequent efflux of lipid from the cells. In this respect, recent studies from other groups have suggested a link between NF-κB/IκBα signalling and cholesterol efflux from macrophages [23]. Inhibition of NF-κB has been shown to increase cholesterol efflux in THP-1 macrophages by upregulating the expression of the ATP-binding cassette transporter [47,48]. Also, blockade of NF-κB activation by overexpression of a degradation-resistant IκBα has been found to increase cholesterol efflux [23]. We have shown previously that the maximum efflux of cholesterol from THP-1 macrophages after lipid loading with CRLPs occurs in the presence of the cholesterol acceptor apoA-I/phosphatidylcholine, which resembles pre-β migrating high-density lipoprotein [49,50]. In the experiments reported here, cholesterol efflux from macrophages in the presence of apoA-I/phosphatidylcholine after loading with CRLPs was strongly affected by the fatty acid composition of the particles, with cholesterol delivered in fish and corn CRLPs effluxed at a considerably faster rate (up to 70% in 24 h) than that from palm and olive CRLPs (28–34% in 24 h) (Fig. 9). Thus, the increased lipid accumulation in macrophages exposed to CRLPs enriched in SFA compared with PUFA observed in our earlier work [30] is caused by a decrease in the efflux of cholesterol as well as an increased rate of uptake. Furthermore, as might be predicted from other studies [23,47,48], the more rapid removal of cholesterol from the cells after loading with CRLPs enriched in PUFA compared with SFA and MUFA was accompanied by a greater inhibition of NF-κB activation. These results, therefore, suggest that the stronger downregulatory effect of CMR enriched in n-3 or n-6 PUFA versus SFA or MUFA on macrophage NF-κB activity plays a role in their relatively decreased induction of lipid accumulation during foam cell formation.

In summary, the studies examining NF-κB binding to DNA and expression of p65–NF-κB and pIκBα reported herein indicate that CRLPs inhibit NF-κB activation in THP-1 macrophages. This conclusion is supported by our demonstration that CRLPs reduce the secretion and mRNA expression of inflammatory cytokines under NF-κB transcriptional control, and downregulate COX-2 mRNA levels in the cells. Furthermore, the effects of CRLPs on NF-κB activation were shown to be modulated by the fatty acid composition of the particles, with CMR enriched in n-3 PUFA, and to a lesser extent n-6 PUFA, having a markedly greater inhibitory effect than those high in SFA or MUFA. Our data also indicate that differential changes in NF-κB activation may play a part in the enhanced induction of macrophage foam cell formation by CMR enriched in n-6 and n-3 PUFA compared with SFA via modulation of the rate of cholesterol efflux from the cells. Overall, this study shows that, despite their induction of foam cell formation, CMR may have protective effects in macrophages culminating in downregulation of inflammatory processes; furthermore, this action depends on the type of dietary fat carried in the particles, with PUFA being more beneficial than SFA or MUFA. These findings provide further evidence for a direct role for CMR in the modulation of atherogenic events in the vasculature.

## Materials and methods

Fetal bovine serum (heat inactivated), penicillin, streptomycin and 2-mercaptoethanol were obtained from Gibco (Paisley, UK). RPMI 1640, Trypan blue, fatty acid-free albumin (BSA), phospholipids, cholesterol, cholesteryl oleate, phorbol 12-myristate 13-acetate, Menhaden fish oil and solid-phase extraction columns (Supelco), SYBR Green JumpStar *Taq* ReadyMix were supplied by Sigma (Poole, Dorset, UK). Palm oil, extra virgin olive oil, corn oil and dried skimmed milk were purchased from domestic suppliers. Phospho-p65–NF-κB (ser536), p65–NF-κB, phospho-IκBα (Ser 32/36) (5A5) and IκBα antibodies were obtained from Cell Signalling Technology (Danvers, MA, USA). Coomassie Plus, bicinchoninic acid-based protein assay kits and horseradish peroxidase conjugate goat anti-(mouse IgG) and anti-(rabbit IgG) (H+L) were supplied by Pierce (Cramlington, UK). RNase Plus extraction kit and Omniscript RT Kit were from Qiagen (Crawley, UK) and ELISA kits for cytokine/chemokine determinations from R&D Systems (Minneapolis, MN, USA). ApoA-I/phosphatidylcholine (molar ratio 1 : 100) [51] was donated by N. Miller (St Bartholomews and the Royal London School of Medicine and Dentistry, London, UK).

### Preparation of CRLPs

Triacylglycerol for the preparation of CRLPs enriched in SFA, MUFA, n-6 PUFA or n-3 PUFA was isolated from palm, olive, corn and fish oil, respectively, as follows: 1.5 mL of each oil was added to 10 mL hexane, 2 mL of the mixture (hexane + oil) was then applied to a solid-phase extraction column (Supelco) previously conditioned with hexane (2 × 2 mL) to remove impurities. After centrifugation (2 min at 2000 ***g***), the eluent containing esterified cholesterol was discarded. Two millilitres of hexane/dichloromethane (9 : 1 v/v) was added to the column and the eluent containing the triacylglycerol was collected after centrifugation (2 min at 2000 ***g***). Triacylglycerol prepared in this way was shown to be uncontaminated with other lipids by TLC in hexane/diethyl ether/formic acid (80 : 20 : 2; v/v/v). Samples were kept under argon at 4 °C until required.

CRLPs were prepared by sonication (power setting 22–24 μm; 20 min at 56 °C) of a lipid mixture containing 70% trilinolein or triacylglycerol from palm, olive, corn or fish oil, 2% cholesterol, 3% cholesteryl ester and 25% phospholipids in 0.9% NaCl (w/v) in Tricine buffer (20 mm, pH 7.4), followed by ultracentrifugation on a stepwise density gradient (2.5 mL d 1.065 g·mL^−1^, 2.5 mL d 1.020 g·mL^−1^, 3 mL d 1.006 g·mL^−1^) at 17 000 ***g*** for 20 min at 20 °C [52]. After removal of the upper layer of grossly emulsified lipids and replacement with an equal volume of NaCl solution (d 1.020 g·mL^−1^), the tubes were centrifuged for 1 h (70 000 ***g***, 20 °C). For apoE binding, lipid particles collected from the top layer were incubated with the dialysed (18 h, 4 °C) d 1.063–1.21 g·mL^−1^ fraction of human plasma (National Blood Transfusion Service, North London Centre, UK) at 37 °C with shaking for 4 h (1 : 2 v/v). CRLPs containing apoE were then isolated by ultracentrifugation at d 1.006 g·mL^−1^ (120 000 ***g***, 12 h, 4 °C), collected from the top layer, purified by a second centrifugation at the same density (202 000 ***g***, 4 h, 4 °C) and stored at 4 °C under argon until required. All preparations were used within 1 week. We have shown previously that CRLPs prepared using these methods contain apoE and no other detectable apolipoproteins [11].

The lipid content of CRLPs (triacylglycerol, total cholesterol and triacylglycerol/total cholesterol) containing trilinolein or triacylglycerol obtained from palm (palm CRLPs) olive (olive CRLPs), corn (corn CRLPs) or fish (fish CRLPs) is shown in Table 1. The small variation in the triacylglycerol and total cholesterol concentrations between the different types of particles are due to the different dilutions of the preparations. There were no significant differences in the triacylglycerol:total cholesterol ratio. In previous studies, we demonstrated that the fatty acid composition of palm, olive, corn and fish oil CRLPs resembles that of their parent oils, so that they are enriched in SFA, MUFA, n-6 PUFA and n-3 PUFA, respectively. In addition, we have shown that they contain similar amounts of apoE [30].

### Culture of THP-1 cells

THP-1 monocytes were maintained in suspension in RPMI 1640 containing 10% fetal bovine serum, penicillin (100 U·mL^−1^), streptomycin (100 mg·mL^−1^) and 2-mercaptoethanol (50 μm) (culture medium) at a density of 3–9 × 10^5^ cells·mL^−1^ at 37 °C in 5% CO_2_/95% air. The cells were induced to differentiate into macrophages by incubation with phorbol 12-myristate 13-acetate (200 ng·mL^−1^) for 72 h. After this time, cells adhering to the culture dishes were washed with warm culture medium to remove any undifferentiated cells and traces of phorbol 12-myristate 13-acetate. The viability of the THP-1 macrophages, as assessed by Trypan blue exclusion, was > 95% in all experiments. Incubation of the cells with CRLPs at a concentration of 0.3 μmol triacylglycerol·mL^−1^ (the maximum used in all experiments) did not significantly affect the viability of the cells as measured by Trypan blue exclusion over the periods tested. In previous studies using a (4,5-dimethylthiazol-2-yl)-2-5-diphenyltetrazolium bromide-based toxicology assay we have also shown that a similar concentration of CRLPs does not cause significant toxicity over a period of 48 h [11]. In all experiments, control macrophages were incubated with a volume of saline (the CRLP vehicle) equal to the volume of CRLPs added to the test incubations.

### Measurement of NF-κB activation

NF-κB activation was measured using a DNA binding assay and a luciferase reporter gene assay. For determination of DNA binding, CRLPs (0.3 μmol triacylglycerol·mL^−1^) were incubated with macrophages (4 ×10^6^ cells·well^−1^) for 6 or 24 h and the cells then washed with NaCl/P_i_ (3 × 3 mL). Nuclear extracts were obtained using a nuclear extraction kit (Active Motif Europe, Rixensart, Belgium) and NF-κB activation measured using a DNA-binding ELISA based kit (TransAM™ NF-κB p65 transcription factor kit, Active Motif) according to the manufacturer’s instructions. For the reporter gene assay, THP-1 macrophages (1 × 10^5^ cells·well^−1^) were transfected with the pNF-κB Luc reporter gene construct (Stratagene, Stockport, UK) using Lipofectamine LTX plus (Invitrogen, Paisley, UK). Sixteen hours after transfection, CRLPs (0.3 μmol·mL^−1^) were added and the incubation was continued for a further 8 h. The cells were then washed with NaCl/P_i_ and lysed using lysis buffer (200 μL·well^−1^) (25 mm glycylglycine, 15 mm MgSO_4_, 4 mm EGTA, 1 mm dithiothreitol and 1% Triton X-100). Lysed cells were centrifuged (5 min, 9000 ***g***) and stored at −80 °C until assayed. Luciferase activity was measured using luciferin (1 mm in glycylglycine buffer, 300 μL·sample^−1^) in a luminometer at 562 nm.

### Immunoblotting procedures

THP-1 macrophages (∼ 3 × 10^6^ cells·dish^−1^) were incubated with CRLPs (0.3 μmol triacylglycerol·mL^−1^) as detailed in the figure legends and expression of p65–NF-κB, phospho-p65–NF-κB, pIκBα and IκBα was determined by immunoblotting. Cell monolayers were washed with NaCl/P_i_ (2 × 4 mL) and whole-cell lysates prepared in lysis buffer [63.5 mm Tris/HCl pH 6.8, 10% glycerol, 2% SDS, 1 mm Na_3_VO_4_, 1 mm 4-(2-aminoethyl) benzenesulfonyl fluoride hydrochloride, 50 μg·mL^−1^ leupeptin, 5%β-mercaptoethanol, and 0.02% bromophenol blue]. Samples were subjected to electrophoresis [Protean II XI (20 cm) electrophoresis system (Bio-Rad)] overnight and then transferred onto poly(vinylidene difluoride) (Immobilon-P) membrane. Membranes were blocked for 3 h in Tris-buffered saline containing Tween-20 (TBST) (50 mm Tris, 150 mm NaCl, and 0.02% v/v Tween-20, pH 7.4) and 5% (w/v) milk powder. For immunodetection of phospho-p65–NF-κB, p65–NF-κB, pIκBα and IκBα, the membranes were incubated overnight in TBST/10% BSA/0.01% sodium azide containing anti-(phospho-p65–NF-κB) IgG, anti-(p65-NF-κB) serum, anti-(pIκBα) IgG or anti-(IκBα) IgG (1 : 1000). Blots were then washed in TBST (8 × 15 min) and incubated with horseradish peroxidase-conjugated rabbit or mouse anti-(rabbit/mouse IgG) as appropriate (1 : 10 000) for 1 h. After further washing (8 × 15 min), immunoreactive bands were visualized by enhanced chemiluminescence (GE Healthcare, Little Chalfont, UK) according to the manufacturer’s instructions [53]. Equal quantities of protein (80 μg·lane^−1^) were loaded, and this was verified by re-probing with antibody recognizing total p65–NF-κB after stripping the membrane in 0.2 m NaOH for 10 min. Band density was analysed using quantity one densitometry software (Bio-Rad) and the intensity of each band was then normalized to the level of total NF-κB. Because total p65–NF-κB is a constitutive protein, it was used for normalization of values for both phospho-p65–NF-kB and pIκBα.

### Production of cytokines

THP-1 macrophages (0.7 × 10^6^ cells·well^−1^) were treated with CRLPs (0.29 μmol triacylglycerol·mL^−1^) for 6, 16 or 24 h. After this time, the medium was removed and centrifuged at 11 337 ***g*** for 10 min prior to cytokine/chemokine analysis. IL-6, IL-1β, TNFα, MCP-1 and TGFβ secretion into the cell culture supernatants were quantified using ELISA kits according to the manufacturer’s instructions.

### Cholesterol efflux measurements

Efflux of CRLP-derived lipid from macrophages was measured as follows: THP-1 macrophages were incubated with CRLPs containing [^3^H]cholesterol for 48 h (30 μg cholesterol·mL^−1^; 4 KBq [^3^H]cholesterol·mL^−1^·L, 52.4 KBq·μmol^−1^) and the medium containing the lipoproteins was then removed. Cells were washed with culture medium (3 × 1 mL) and incubations continued in fetal bovine serum-free culture medium for 24 h in the presence of ApoA-I/phosphatidylcholine (100 μg·mL^−1^). At the times indicated in the text, aliquots of the medium were taken and the radioactivity was assayed by liquid scintillation counting. The cells were washed with NaCl/P_i_ (3 × 3 mL), resuspended in 500 μL NaOH (0.5 m), and cell-associated radioactivity determined.

### mRNA analysis

THP-1 macrophages (1.5 × 10^6^ cells·well^−1^) were incubated with CRLPs (0.3 μmol triacylglycerol·mL^−1^) for 16 or 24 h. Total RNA was extracted using an RNAeasy Plus Mini Kit (Qiagen), and the abundance of transcripts for TNFα, IL-6, IL-1β, MCP-1, TGFβ, COX-2, GAPDH and β-microglobulin were determined by quantitative real-time PCR. The reverse transcription reaction was carried out using an Omniscript RT kit (Qiagen) according to the manufacturer’s instructions. cDNA was amplified using an Opticon 2 DNA Engine and a SYBR Green quantitative real-time PCR kit (Sigma, Gillingham, UK) and the forward and reverse primers shown in Table 2. The conditions were as follows: denaturation at 94 °C for 2 min, followed by amplification (94 °C, 15 s), annealing for 1 min at the temperature shown in Table 1 and extension (72 °C for 1 min) for 37 cycles; and finally a melting curve programme (60–95 °C, rate of 0.2 °C·s^−1^). The Ct values were determined by automated threshold analysis using opticon monitor 2 software. Data were normalized using the values obtained for GAPDH (COX-2) or β-microglobulin (all other genes). The fold change in mRNA expression in CRLP-treated compared with control cells was calculated as described by Pfaffl [54].

**Table 2:** Primer sequences and annealing temperatures for quantitative real-time PCR. COX, cyclooxygenase; IL, interleukin; MCP-1, monocyte chemoattractant protein-1; TGFβ, transforming growth factor β; TNFα, tumour necrosis factor α.

Gene product	Forward	Reverse	Annealing temperature (°C)
TNFα			56.5
IL-6			56.5
IL-1β			59.0
MCP-1			59.0
TGFβ			57.5
COX-2			61.1
GAPDH			58
β-microglobulin			57.0

### Other analytical methods

Total IκB in THP-1 macrophages was determined by ELISA according to the manufacturer’s instructions using a kit supplied by Assay Designs (Ann Arbor, MI, USA). The total cholesterol and triacylglycerol content of CRLPs were determined by enzymatic analyses using commercially available kits (Alpha Laboratories, Eastleigh, UK). Cell protein contents were measured by the method of Bradford [55] except for those in the whole cell lysates employed for immunoblotting which were quantified using the bicinchoninic acid protein assay.

### Statistical analysis

Data were analysed by one-way ANOVA followed by Tukey’s test (single time point) or two-way ANOVA followed by Bonferroni’s multiple comparison test (multiple time points), except where indicated otherwise.

## Abbreviations

apo: apolipoprotein
CMR: chylomicron remnants
COX: cyclooxygenase
CRLPs: chylomicron remnant-like particles
IL: interleukin
IκB: inhibitor of κB
MCP-1: monocyte chemoattractant protein-1
MUFA: monounsaturated fatty acids
NF-κB: nuclear factor-κB
oxLDL: oxidized low density lipoprotein
PUFA: polyunsaturated fatty acids
SFA: saturated fatty acids
TGFβ: transforming growth factor β
TNFα: tumour necrosis factor α
